# Identification of New Potential LncRNA Biomarkers in Hirschsprung Disease

**DOI:** 10.3390/ijms21155534

**Published:** 2020-08-02

**Authors:** Ana Torroglosa, Leticia Villalba-Benito, Raquel María Fernández, Berta Luzón-Toro, María José Moya-Jiménez, Guillermo Antiñolo, Salud Borrego

**Affiliations:** 1 Department of Maternofetal Medicine, Genetics and Reproduction, Institute of Biomedicine of Seville (IBIS), University Hospital Virgen del Rocío/CSIC/University of Seville, 41013 Seville, Spain; ana.torroglosa@juntadeandalucia.es (A.T.); leticia.villalba.benito@hotmail.es (L.V.-B.); raquelm.fernandez.sspa@juntadeandalucia.es (R.M.F.); berta.luzon@ciberer.es (B.L.-T.); guillermo.antinolo.sspa@juntadeandalucia.es (G.A.); 2 Centre for Biomedical Network Research on Rare Diseases (CIBERER), 41013 Seville, Spain; 3 Department of Pediatric Surgery, University Hospital Virgen del Rocío, 41013 Seville, Spain; mariajosemoyajimenez@gmail.com

**Keywords:** gastrointestinal tract, Hirschsprung disease, enteric nervous system, stem cells, neural crest cells, enteric precursor cells, epigenetic mechanisms, long noncoding RNA

## Abstract

Hirschsprung disease (HSCR) is a neurocristopathy defined by intestinal aganglionosis due to alterations during the development of the Enteric Nervous System (ENS). A wide spectrum of molecules involved in different signaling pathways and mechanisms have been described in HSCR onset. Among them, epigenetic mechanisms are gaining increasing relevance. In an effort to better understand the epigenetic basis of HSCR, we have performed an analysis for the identification of long non-coding RNAs (lncRNAs) by qRT-PCR in enteric precursor cells (EPCs) from controls and HSCR patients. We aimed to test the presence of a set lncRNAs among 84 lncRNAs in human EPCs, which were previously related with crucial cellular processes for ENS development, as well as to identify the possible differences between HSCR patients and controls. As a result, we have determined a set of lncRNAs with positive expression in human EPCs that were screened for mutations using the exome data from our cohort of HSCR patients to identify possible variants related to this pathology. Interestingly, we identified three lncRNAs with different levels of their transcripts (*SOCS2-AS*, *MEG3* and *NEAT1*) between HSCR patients and controls. We propose such lncRNAs as possible regulatory elements implicated in the onset of HSCR as well as potential biomarkers of this pathology.

## 1. Introduction

Hirschsprung disease (HSCR:OMIM 142623), or congenital megacolon, is the most common neurocristopathy in humans, and it is considered a rare disease with an incidence of ~1/5000 live births [1]. HSCR is characterized by the absence of the enteric ganglia due to a failure in proliferation, survival, migration and/or differentiation of the enteric neural crest cells (ENCCs) avoiding the colonization of the distal intestine [2]. Up to 5–20% of cases have been described to be familial with either an autosomal dominant or recessive pattern of inheritance, though most of cases are sporadic, showing a complex inheritance pattern with low, sex-dependent penetrance and variable expression. HSCR mainly appears as isolated forms (70%), and the remaining cases (30%) present with other clinical manifestations (syndromic HSCR). Based on the length of the affected region, different phenotypes have been established: short-segment (S-HSCR), where aganglionosis is limited up to the upper sigmoid colon, and long-segment (L-HSCR), when the aganglionosis exceeds the splenic flexure, including the total colonic aganglionosis (TCA) and total intestinal aganglionosis (TIA) forms [3].

For most isolated and sporadic forms, a complex genetic basis has been proposed, where the presence of several genetic variants acting in an additive or multiplicative manner leads to the disease [4]. The *RET* proto-oncogene (OMIM 164761) is the main gene associated with HSCR [5,6,7], although there are many other genes related to the disease, among which most are involved in the development of the Enteric Nervous System (ENS) [8].

The development of ENS is a process highly regulated by a large number of molecules, signaling pathways and different mechanisms. As a consequence, ENS formation requires an exhaustive control at those different levels, and alterations at any of them may result in the onset of HSCR. In this sense, the epigenetic mechanisms play an important role on the establishment of the specific gene expression patterns in many biological processes, constituting an emerging research area in the study of ENS development and specifically in HSCR [9].

Epigenetic processes are defined as “the structural adaptation of chromosomal regions so as to register, signal or perpetuate altered activity state” [10]. Changes at this level are mainly stable but, although they are transmitted along generations, the relevant influence of the environment has also been shown [11]. Among the different epigenetic mechanisms (DNA methylation, histone modifications, polycomb repression, ATP-dependent chromatin remodeling, non-coding RNA) [10,12,13,14,15], this study is focused on the analysis on non-coding RNA, specifically long non-coding RNAs (lncRNAs). LncRNAs are transcripts with a length greater than 200 nucleotides and carry out a crucial regulatory role on gene expression at different levels (epigenetic, transcriptional, translational, post-transcriptional and post-translational) in many biological processes through different mechanisms (interacting with mRNA, DNA, protein and miRNA) [13,16]. These molecules have been widely related to different pathologies, especially in cancer, and in this case it is worth noting those that affect the nervous system, as is the case of the ENS that results in the appearance and progression of HSCR. These molecules have acquired an important role as biomarkers in this pathology, hence the interest in studying them [9,17,18,19,20].

Enteric precursor cells (EPCs) isolated from human postnatal intestinal tissue is a robust tool for studying the development of ENS. These cells grow in clusters known as neurosphere-like bodies (NLBs) and include stem cells with their progeny derived from the neural crest. It has been described that the EPCs contained in the NLBs can be transplanted into the aganglionic intestine to restore their contractile properties [21,22]. In addition, in previous studies we validated EPC cultures for the study of the ENS and HSCR through different methodological approaches [23,24]. Therefore, the use of human EPCs is a “more physiological” tool than cell lines and a better system than gut tissue to study the regulatory mechanisms and implicated molecules during embryonic ENS development.

It would be worthy to note that the current tests for the diagnosis of HSCR have both advantages and disadvantages in availability, technique difficulty, radiation exposure and invasiveness [25]. In this sense, new diagnostic tools, such as the use of biomarkers, are being developed to try to solve such inconvenience, lncRNAs being one of the molecules analyzed in HSCR context [26].

In this study, unlike other previous assays, we have used EPCs from HSCR patients and controls for the first time to perform an analysis by qRT-PCR of a set of 84 lncRNAs, in order to identify new lncRNAs associated with ENS development and to evaluate their potential role in this pathology.

## 2. Results

### 2.1. LncRNA in Human EPCs

**Figure 1:**
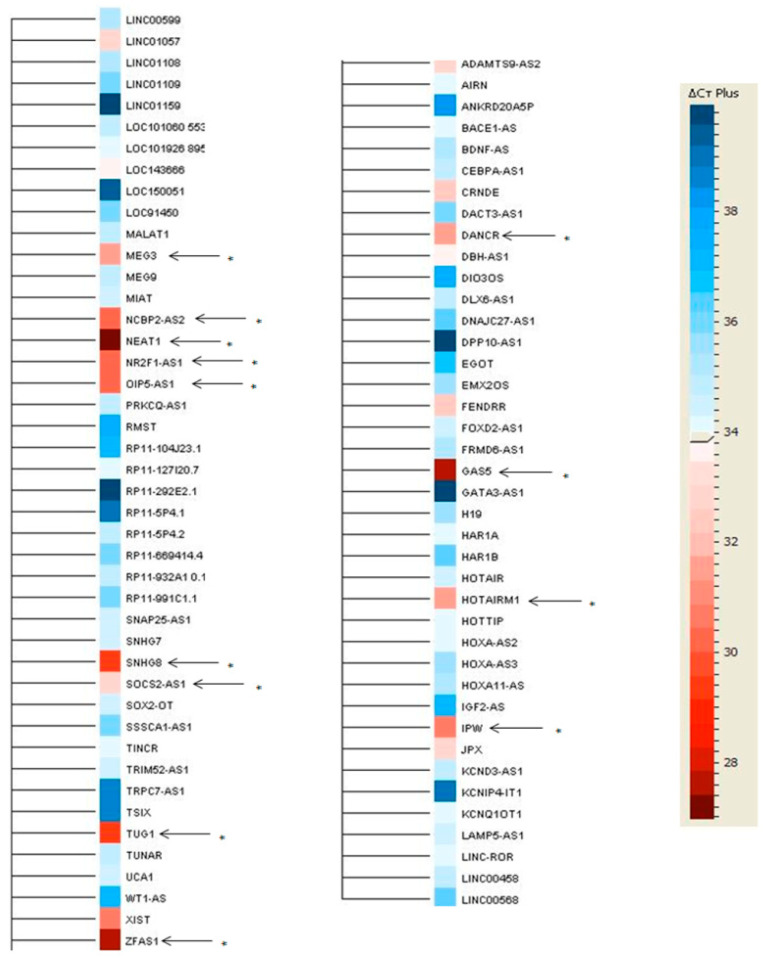
Long non-coding RNAs (LncRNAs) significantly present in human enteric precursor cells (EPCs). Representative heat map of the qRT-PCR experiments in human EPCs. The heat map shows the positive (red) or negative (blue) expression levels of lncRNA in human EPCs. Arrow and asterisk indicate the 13 lncRNA with a statistically significant presence. The color scale represents the Δ*C*t plus, shown on the right side. Cycle threshold (*C*t) = 34.

From the 84 lncRNAs analyzed with the Human Cell Development & Differentiation RT2 lncRNA PCR Array, 13 of them (*DANCR, GAS5, IPW, HOTAIRM1, MEG3, NEAT1, NR2F1-AS1, NCBP2-AS2, OIP5- AS1, SNHG8, TUG1, ZFAS1, SOCS2-AS1*) showed a presence of their transcripts (Figure 1) in human EPCs (Table 1). Except for *MEG3*, all the remaining lncRNAs had not been previously described to be associated to HSCR. This result led us to consider that these 13 lncRNAs could play a role in ENS formation and therefore in HSCR.

### 2.2. LncRNAs with Differential Leves in EPCs from HSCR Patients

**Figure 2:**
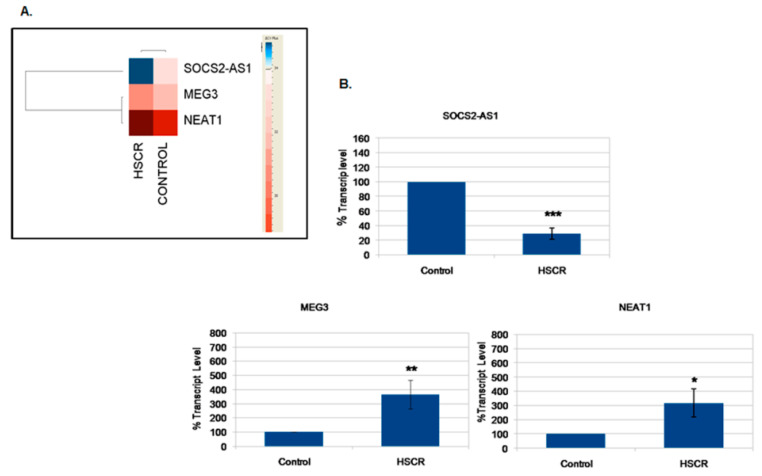
LncRNAs with different transcript levels in Hirschsprung disease enteric precursor cells (HSCR-EPCs)**.** (**A**) The Heat map represents the differential transcript levels of the lncRNA in HSCR-EPCs, the upregulation (red) and downregulation (blue) are shown. The color scale indicates the Δ*C*t plus, shown on the right side. *C*t = 34. (**B**) Graphics show the percentages of transcript level of each lncRNA (*SOC2-AS1, MEG3* and *NEAT1*) in EPCs from HSCR patients and controls. * *p* value < 0.05, ** *p* value < 0.01 and *** *p* value < 0.001.

Among the 13 lncRNAs identified in the human EPCs (controls), we wanted to look for possible alterations at their transcript levels in the EPCs from HSCR patients (HSCR-EPCs). With this aim, we performed a comparative qRT-PCR analysis between EPCs from HSCR patients and controls. Three of them showed a different profile between HSCR patients and controls (*SOCS2-AS1, MEG3* and *NEAT1*) (Figure 2).

### 2.3. Identification of lncRNA Variants in the Exomes from HSCR Patients

To evaluate the involvement of these molecules in HSCR by alternative mechanisms other than their expression level, the search of potential pathogenic sequence variants was performed in the lncRNAs expressed in EPCs, through the analysis of the Whole Exome Sequencing (WES) data from 56 HSCR patients from our cohort. As a result, a group of rare variants with minor allele frequency (MAF) ≤ 0.01 in *MEG3*, *NEAT1* and *ZFAS1* was identified in four different unrelated patients (Table 2). The remaining variants are available under request.

## 3. Discussion

Much evidence suggests a relationship between lncRNAs and HSCR, all based on the comparative expression assays of specific lncRNAs in bowel tissue from HSCR patients and controls [73,74,75,76,77,78]. In our study we have performed a differential qRT-PCR assay in the EPCs from HSCR patients and controls, which has allowed us to identify new lncRNAs as potential regulatory elements implicated in ENS development and HSCR onset.

Among the 13 identified lncRNAs in human EPCs, 10 of them (*DANCR, GAS5, IPW, HOTAIRM1, NR2F1-AS1, NCBP2-AS2, OIP5-AS1, SNHG8, TUG1* and *ZFAS1*) did not show differential levels in the HSCR-EPCs. Despite this fact, most of them have been related to crucial cellular processes for ENS formation (proliferation, survival, migration and differentiation) [27,31,35,50,53,57,60,64]. In addition, *TUG1* is a lncRNA that binds to Polycomb Repressive Complex 1 and 2 (PRC 1 and 2) [62,63]. In this sense, *AEBP2* and *EED* are components of PCR2, and both proteins have been related to HSCR. The heterozygous *Aebp2* mutant mice showed HSCR-like phenotype, while *EED* was observed to be upregulated in HSCR patients [79,80]. Taking all this information into account, such lncRNAs might be considered as regulatory elements in ENS development and could be implicated in the onset of HSCR, although additional studies are needed to demonstrate this hypothesis.

More interestingly, *SOCS2-AS1, MEG3* and *NEAT1* showed different transcript levels between EPCs from HSCR patients and controls. Particularly, *SOCS2-AS1* was significantly downregulated in HSCR patients. This lncRNA has been described in relation with prostate cancer and retinal Müller cell immune responses and has also been identified as a biomarker in coronary artery disease [67,68,69]. In this sense, *SOCS2-AS1* would be a good candidate for further consideration as a gene related to the onset of HSCR and, therefore, of great interest to continue investigating in this line.

Regarding *MEG3* (significantly upregulated in our study), it has already been described as a potentially related lncRNA with HSCR. In contrast with our results, in another study it was downregulated in gut tissue from HSCR patients, although it was not specified if authors used ganglionic or aganglionic tissues [40]. In our study we have used HSCR-EPCs from the ganglionic gut region of HSCR patients, which could explain such different outcomes.

In addition, we have identified two variants n.1242-2384C > T (rs11624207) and n.1183 + 41G > T (rs147149937) in *MEG3* in two different S-HSCR patients. Both patients also carried variants in specific regions (downstream and intergenic) of another identified lncRNA in human EPCs, *ZFAS1* (n.*47A > C and c.1050-176_1050-174delATA respectively). Moreover, a variant was previously located in one of these patients in a HSCR-related gene, *NTF3* (c.226G > A; G76R) [81]. These variants alone do not justify the HSCR phenotype, but our results (different transcript levels/sequence variants) may point out the implication of *MEG3* in ENS development and the onset of HSCR.

Finally, *NEAT1* was upregulated in our study, and a previously not described variant (n.*4662_*4663insG) was identified in its sequence in one S-HSCR patient. This patient carries another variant in a HSCR-related gene, *EDNRB* (c.466C > T; P156S) [82]. Specifically, this lncRNA has been widely related with cancer and, to a lesser extent, with neurogenesis [45]. This process, which involves the generation of neurons, is essential for the correct formation of ganglions during ENS development. Therefore, all this evidence leads us to suggest that *NEAT1* may have a role in the HSCR context.

In summary, we report here the presence of a set of lncRNAs in human EPCs, as well as propose the possible role of two of them (SOX2-AS1 and NEAT1) in the initiation of HSCR. Furthermore, our results support the involvement of *MEG3* in this pathology. Numerous studies about lncRNAs as biomarkers for the molecular diagnosis of different human diseases can be found in the existing bibliography. In this sense, *SOCS2-AS1* and *NEAT1* may serve as new biomarkers for molecular diagnosis in this pathology, as well as *MEG3* previously related with HSCR. It is interesting to highlight that this study has contributed to the knowledge of the epigenetic basis of HSCR and has again shown the important role that epigenetic regulators play in the development of ENS and the initiation of HSCR.

## 4. Material and Methods

### 4.1. Enteric Precursor Cells Culture Obtained from Human

Enteric precursor cells were extracted from human postnatal tissues of the ganglionic gut region from 16 sporadic, non-related patients diagnosed with HSCR (2 L-HSCR, 13 S-HSCR, 1 TCA; 12 male, 4 female) as well as from 5 controls (3 male, 2 female) who were patients without ENS alteration, and the gut resection was necessary (anorectal malformations). The isolation of EPCs from both types of patients was performed following the protocol established by Ruiz-Ferrer et al. [23]. Human EPCs were cultured as neurosphere-like bodies. From all the human participants or their guardians, written informed consent for surgery, clinical and molecular genetic studies was obtained. The Ethics Committee for Research of the University Hospital Virgen del Rocío, Seville, Spain, approved this study (Project identification code: 1509-N-16 (December 2015), 2149-N-19 (December 2019) and 20191220134633-1 (October 2019, which complies with the tenets of the declaration of Helsinki.

### 4.2. Analysis of lncRNA Expression in Human EPCs

The qRT-PCR assay was used to quantify the expression level of the lncRNAs from human EPCs. RNA was isolated and cDNA was synthesized using µMACS mRNA Isolation Kit and µMACS cDNA Synthesis Kit in a thermo MAKSTM Separator (MACS Miltenyi Biotech, Bergisch Gladbach, Germany) and RNAeasy Micro kit and RT^2^ First Strand kit (Qiagen, Dusseldorf Germany). Expression study was performed on RT^2^ lncRNA PCR Array Human Cell Development & Differentiation using individual assays of the selected lncRNAs (Qiagen, Dusseldorf, Germany)) in an Applied Biosystems 7900HT and 7500HT systems respectively (Life Technologies, USA) with Sybr green method. Data analysis was carried out using the RQ Manager Software (Life Technologies, New York, NY, USA). *ACTB* or *GAPDH* was used as endogenous control. Following the software recommendations, the upper limit of the cycle threshold (Ct) was set to 34. We considered positive expression exclusively when Ct values were lower than such value.

### 4.3. Analysis of lncRNA Coding Sequence in HSCR Patients

The sequence of lncRNAs expressed in human EPCs was analyzed in the exomes from 56 HSCR patients (39 sporadic and 17 familial cases) for the identification of variants related to this pathology [83]. The resulting variants in relation with HSCR (Single Nucleotide Variants, SNVs, and short insertions or deletions, Indels) were annotated using Annovar (hg19 Refgene) (http://wannovar.wglab.org/index.php). The MAF value of the variants was searched in a Spanish population variant server web page (CIBERER Spanish Variant Server, CSVS, publicly available http://csvs.babelomics.org/) [84].

### 4.4. Statistical Analysis

Expression data are presented as the mean ± SEM (Standard Error Mean) of values obtained from at least three experiments. Comparisons between values of expression level obtained in EPCs from controls and HSCR patients were analyzed using the Student’s *t* test. Differences were considered significant when *p* value < 0.05.

## Figures and Tables

**Table 1:** LncRNAs identified in human EPCs.

LncRNA	Function	Associated Diseases	Bibliography
*DANCR*	Negative regulator of cell differentiation	Bone Disease and Osteoporosis.	[27,28,29,30]
*GAS5*	Cellular growth arrest and apoptosis/Embryonic development	Inflammatory Bowel Disease and Autoimmune Disease.	[31,32,33,34]
*IPW*	Role in the imprinting process/Differentiation and development	Prader–Willi Syndrome and Chromosomal Disease.	[35,36]
*HOTAIRM1*	Cell-fate programming and reprogramming	Leukemia and Pancreatic Ductal Adenocarcinoma.	[37,38,39]
*MEG3*	Cell-fate programming and reprogramming/Mesenchymal stem cells and osteoblast differentiation/Skeletal muscle development	Hirschsprung disease, Kagami–Ogata Syndrome and Functionless Pituitary Adenoma.	[40,41,42,43]
*NEAT1*	Transcribed from the multiple endocrine neoplasia locus/Skeletal muscle development/Embryonic stem cell pluripotency and differentiation/Neurogenesis	Dengue Disease and Relapsing-Remitting Multiple Sclerosis.	[44,45,46,47,48,49]
*NR2F1-AS1*	Promoted cell proliferation and migration/Embryonic development	Rhabdomyosarcoma and Hepatocellular Carcinoma.	[50,51,52]
*OIP5-AS1*	Maintains cell proliferation in embryonic stem cells/Neurogenesis	Hepatoblastoma and Glioma.	[53,54,55,56]
*SNHG8*	Cellular growth and migration/Neurogenesis	Epstein–Barr Virus-Associated Gastric Carcinoma and Malignant Pleural Mesothelioma.	[57,58,59]
*TUG1*	Epigenetic regulation of transcription through interaction with the polycomb repressor complex/Embryonic stem cell pluripotency and differentiation/Neurogenesis	Intrahepatic Cholangiocarcinoma and Relapsing-Remitting Multiple Sclerosis.	[60,61,62,63]
*ZFAS1*	Differentiation and development	Breast Ductal Carcinoma and Rheumatoid Arthritis.	[64,65,66]
*SOCS2-AS1*	Neurogenesis	Prostate Cancer.	[67,68,69]
*NCBP2-AS2*	Embryonic development	Lung cancer and Osteoporosis	[70,71,72]

**Table 2:** Sequence variants (MAF ≤ 0.01) determined in lncRNAs identified in human EPCs.

Patient ID	Genes	RefSeq	Variants (Genomic Location)	Variants (Gene Location)	rs	Phenotype	Variants in Other HSCR-Genes
8079	*MEG3*	NR_002766.1	chr14: g.101324644C > T	n.1242-2384C > T	rs11624207	S-HSCR	-
	*ZFAS1*	NR_003604.2	chr20: g. 47905844 A > C	n.*47A > C (downstream)	-		-
4217	*ZFAS1,KCNB1*	XM_001716063.1	chr20: g.47956681_47956683delATA	c.1050-176_1050-174delATA (intergenic)	-	S-HSCR	*NTF3* (NM_002527) c.226G > A (rs540320780)
	*MEG3*	NR_002766.2	chr14: g.101302678G>T	n.1183 + 41G >T (intronic)	rs147149937		
16987	*NEAT 1*	NR_002802.1	chr11: g.65211817_65211818insG	n.*4662_*4663insG	-		*EDNRB:* (NM_000115) c.466 C > T (CM100206/7)
4678	*ZFAS1,KCNB1*	XM_001716063.1	chr20: g.47956675_47956683delATAATAATA	c.1050-182_1050-174delATAATAATA (intergenic)	rs530512526	S-HSCR	-

## Abbreviations

LncRNA	Long non-coding RNA
ENS	Enteric Nervous System
EPCs	Enteric Precursor Cells
HSCR	Hirschsprung disease
L-HSCR	Long-segment HSCR
S-HSCR	Short-segment HSCR
TCA	Total Colonic Aganglionosis
TIA	Total Intestinal Aganglionosis
ENCCs	Enteric Neural Crest Cells
PRC	Polycomb Repressive Complex
qRT-PCR	Quantitative real-time PCR
mRNA	Messenger RNA
Ct	Cycle Threshold
ΔCt Plus	Delta Ct Plus global control mean
WES	Whole Exome Sequencing
MAF	Minor Allele Frequency
SNVs	Single Nucleotide Variants
Indels	Short Insertions or Deletions
